# Dietary Natural Products for Prevention and Treatment of Breast Cancer

**DOI:** 10.3390/nu9070728

**Published:** 2017-07-08

**Authors:** Ya Li, Sha Li, Xiao Meng, Ren-You Gan, Jiao-Jiao Zhang, Hua-Bin Li

**Affiliations:** 1 Guangdong Provincial Key Laboratory of Food, Nutrition and Health, Department of Nutrition, School of Public Health, Sun Yat-Sen University, Guangzhou 510080, China; liya28@mail2.sysu.edu.cn (Y.L.); mengx7@mail2.sysu.edu.cn (X.M.); zhangjj46@mail2.sysu.edu.cn (J.-J.Z.); 2 School of Chinese Medicine, Li Ka Shing Faculty of Medicine, The University of Hong Kong, Hong Kong 999077, China; 3 School of Biological Sciences, The University of Hong Kong, Hong Kong 999077, China; ganry@connect.hku.hk; 4 South China Sea Bioresource Exploitation and Utilization Collaborative Innovation Center, Sun Yat-Sen University, Guangzhou 510006, China

**Keywords:** breast cancer, soy, fruit, vegetable, anticancer, mechanism of action

## Abstract

Breast cancer is the most common cancer among females worldwide. Several epidemiological studies suggested the inverse correlation between the intake of vegetables and fruits and the incidence of breast cancer. Substantial experimental studies indicated that many dietary natural products could affect the development and progression of breast cancer, such as soy, pomegranate, mangosteen, citrus fruits, apple, grape, mango, cruciferous vegetables, ginger, garlic, black cumin, edible macro-fungi, and cereals. Their anti-breast cancer effects involve various mechanisms of action, such as downregulating ER-α expression and activity, inhibiting proliferation, migration, metastasis and angiogenesis of breast tumor cells, inducing apoptosis and cell cycle arrest, and sensitizing breast tumor cells to radiotherapy and chemotherapy. This review summarizes the potential role of dietary natural products and their major bioactive components in prevention and treatment of breast cancer, and special attention was paid to the mechanisms of action.

## 1. Introduction

Globally, breast cancer is the most commonly diagnosed cancer and the major cause of cancer-related death among females [1]. In the United States alone, there are 255,180 new cases of breast cancer and 41,070 deaths projected to occur in 2017 [2]. Breast cancer is generally categorized into estrogen receptor (ER)-positive (such as MCF-7 and T47D cell lines) and ER-negative (such as MDA-MB-231, MDA-MB-468, SKBR3 and MDA-MB-453 cell lines) breast cancer. By using more biomarkers such as progesterone receptor (PR), and human epidermal growth factor receptor 2 (HER2), breast cancer is further divided into several molecular subtypes, such as luminal A, luminal B, basal-like and HER2-positive ones [3,4]. Basal-like breast cancer is also considered as triple-negative breast cancer (TNBC) in some cases, because TNBC is characterized as lacking the expression of these three biomarkers [5]. These distinct subtypes of breast tumor would response differently to treatment, which made breast cancer extremely intractable. Currently, surgical resection, adjuvant chemotherapy, radiotherapy and hormone therapy represent the main treatment options for early-stage breast cancer [6]. However, the development of drug resistance and major side effects has weakened the efficacy of these therapies [7,8]. Besides, triple-negative breast cancer does not respond to hormone therapy [9]. This situation urges the research of finding more effective prevention and treatment strategies with fewer side effects for breast cancer. Many exogenous and endogenous factors could affect the onset and development of breast cancer. Exogenous factors include reproductive, environmental and lifestyle factors, such as early menarche [10], nulliparity, oral contraceptive use [11], parity and lactation (never having or short duration of breast feeding) [12,13], use of hormone replacement therapy [14], alcohol consumption [15], diabetes [16,17], obesity [18], and night work (circadian disruption) [19]. Genetic risk factors, such as mutations on breast cancer susceptibility gene 1 (BRCA1) and BRCA2, only account for approximately 5–10% of all breast cancer incidences [20]. Therefore, the prevention of breast cancer is highly crucial.

**Figure 1:**
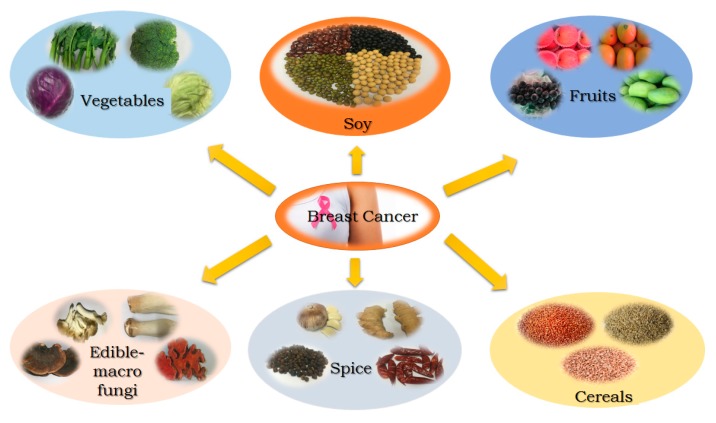
Dietary natural products that showed inhibitory effects on breast cancer.

Diet and nutrition have been considered as an effective preventive strategy for cancer. A bunch of dietary natural products have shown a potential role in prevention and treatment of cancers [21,22,23,24,25,26]. A recently published meta-analysis, which included 93 studies, pointed out that results on breast cancer were among the few reaching a convincing evidence of a protective effect of healthy dietary pattern and cancer risk, and the effect was especially prominent in postmenopausal, hormone receptor–negative women [27]. Furthermore, various epidemiological studies suggested that consumption of soy products, fruits, and vegetables (especially cruciferous vegetables) are associated with reduced risk of breast cancer [28,29], and high consumption of some dietary natural products might reduce the recurrence and increase the survival rate of breast cancer [30,31]. In addition, experimental studies indicated that many dietary natural products and their bioactive components showed inhibitory effects on breast cancer (Figure 1), through downregulating ER-α expression and activity, inhibiting proliferation, metastasis and angiogenesis of breast tumor cells, inducing apoptosis and cell cycle arrest, and sensitizing breast tumor cells to radiotherapy and chemotherapy [32,33,34,35]. Therefore, use of naturally occurring dietary substances could be a practical approach to prevention and treatment of breast cancer [36]. The objective of this review is to summarize the role of dietary natural products and their bioactive compounds in the prevention and treatment of breast cancer, and discuss the mechanisms of action.

## 2. Soy

Soy products have been widely consumed in Asian regions for centuries. Many potential health benefits have been linked with intake of soy products, such as lower incidences of coronary heart diseases [37], type 2 diabetes [38] and breast cancer [39]. Soy products are rich in isoflavones, and a meta-analysis of prospective studies indicated that intake of isoflavones was nearly significantly associated with decreased risk of breast cancer [40]. Besides, another meta-analysis, which included five cohort studies, found that post-diagnosis intake of soy food was associated with reduced mortality (HR for highest vs. lowest dose was 0.84, 95% CI = 0.71–0.99) and recurrence (HR = 0.74, 95% CI = 0.64–0.85) of breast cancer, indicating that soy food intake might be linked with better survival [41]. Indeed, numerous studies have supported the beneficial role of soy food intake for patients with breast cancer, although some reverse results were also observed. Given the vast number of studies, relevant peer-reviewed articles published in English within 5 years were included and discussed in this section.

### 2.1. Epidemiological Evidence

Most epidemiological studies have supported an inverse relationship between soy consumption and risk of breast cancer, though most studies were conducted in Asian population due to the different food preferences [39,42,43]. In recent years, epidemiological studies have widely investigated the effects of soy foods on breast cancer defined by different status. The amount of soy isoflavone consumption, estrogen receptor status of tumor, menopausal status of patients and timing of dietary exposure, could influence the soy-breast cancer association [44]. A recent study found an inverse association between adult soy intake and breast cancer risk (HR for fifth versus first quintile soy protein intake = 0.78; 95% CI = 0.63–0.97) of the population based in Shanghai Women’s Health Study, with a predominance observed in premenopausal women (HR = 0.46; 95% CI: 0.29–0.74). Further stratified analyses found that soy intake during adulthood was significantly associated with decreased risk of ER−/PR− breast cancer in premenopausal women (HR = 0.46; 95% CI = 0.22–0.97) and decreased risk of ER+/PR+ breast cancer in postmenopausal women (HR = 0.72; 95% CI = 0.53–0.96). The HER2 status did not show a significant influence on the association [45]. Likewise, data extracted from the Takayama study in Japan pointed out that the relative risk of postmenopausal breast cancer was lower in women with larger consumption of soy (trend *p* = 0.023) and isoflavone (trend *p* = 0.046), although intake of soy and isoflavone did not affect the relative risks of premenopausal breast cancer [46]. In addition, it was found that high soy protein intake was associated with decreased breast cancer death (HR = 0.71, 95% CI = 0.52–0.98). Further stratified analysis pointed out that high intake of soy isoflavone was associated with a better prognosis of ER positive breast cancer (HR = 0.59, 95% CI = 0.40–0.93) [47]. Furthermore, data from the Korean Hereditary Breast Cancer Study reported that intake of soy products showed a lower risk of breast cancer in BRCA2 mutation carriers (HR: 0.39; 95% CI = 0.19–0.79 for the highest quartile) than noncarriers [48]. Besides, a study investigated the association between soy intake and tumor tissue miRNA and gene expression of TNBC patients, and found that long-term prediagnosis soy intake might be associated with elevated expression of tumor suppressors (such as miR-29a-3p and insulin-like growth factor 1 receptor (IGF1R)), and declined expression of oncogenes [49]. Ethnic diversity could also affect the soy-breast cancer association, thus some studies included participants from different ethnic groups or combined data from different cohort studies. A study combined 9514 breast cancer survivors from 2 US cohorts and 1 Chinese cohort. It was suggested that postdiagnosis soy food consumption (≥10 mg isoflavones/day) was related to a significant decreased risk of recurrence (HR = 0.75; 95% Cl = 0.61–0.92) and a nonsignificant lower risk of breast cancer specific mortality [30].

However, controversy still exists in this topic whether soy intake is associated with a reduced breast cancer risk. A multiethnic cohort study recruited women of African Americans, Latinos, Japanese Americans, Caucasians, and Native Hawaiians, and found that intake of prediagnosis soy was not associated with all-cause or breast cancer specific mortality [50]. Furthermore, no statistically significant relationship was observed between dietary isoflavone intake and overall risks of breast cancer across racial/ethnic groups in the same cohort [51]. Besides, a study showed that total soy food intake was not associated with an increased risk of cancer recurrence, but high intake of soy isoflavone increased the risk of cancer recurrence in HER2-positive breast cancer patients [52]. Moreover, according to a randomized phase II trial, a 6-month intervention of mixed soy isoflavones in high-risk or healthy adult Western women induced no reduction of breast epithelial proliferation, suggesting the poor efficacy of soy isoflavones for breast cancer prevention and an even possible adverse effect on premenopausal women [53]. The inconsistence of these results might be attributed to multiple reasons, such as the difference between food frequency questionnaires (FFQ) designed to capture the amount of soy intake [54].

### 2.2. Experimental Evidence

Despite the inconsistency in the abovementioned epidemiological studies, the fact that prevalence of breast cancers in Asia is much lower than that in North American and European countries has still raised an increasing interest in soy isoflavones as a potential therapeutic agent in breast cancer chemoprevention [55]. In fact, numerous experimental studies have demonstrated the inhibitory effects of soy products and soy isoflavones on breast cancer through various mechanisms of action.

**Figure 2:**
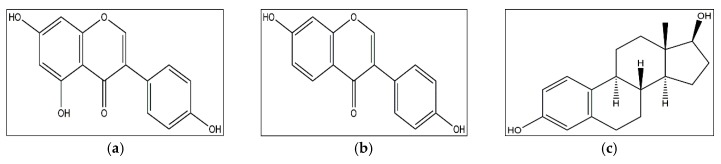
Structures of genistein (**a**); daidzein (**b**); 17-β-estradiol (**c**).

In general, soy isoflavones (such as genistein and daidzein) exert their anti-breast cancer effects through the ER-dependent signaling pathways, due to the structural resemblance with 17-β-estradiol (Figure 2). For instance, genistein, one of the predominant soy isoflavones, could bind with both ERα and ERβ [56,57]. The ERα/ERβ ratio is a prognostic marker for breast tumors, and plays a vital role in the effect of soy isoflavones on breast cancer cells [58,59,60]. Genistein regulated the proliferation and mitochondrial functionality of breast cancer cells in an ERα/ERβ ratio-dependent way, since genistein treatment induced cell cycle arrest and improved mitochondrial functionality in T47D cells (low ERα/ERβ ratio), without affecting MCF-7 (high ERα/ERβ ratio) and MDA-MB-231 (ER-negative) cells [58]. Likewise, genistein regulated oxidative stress, uncoupling proteins, antioxidant enzymes and sirtuin, which was also dependent on ERα/ERβ ratio of breast cancer cells [59]. Furthermore, the synergetic effect of genistein with other anticancer drugs (cisplatin, paclitaxel or tamoxifen) was also dependent on ERα/ERβ ratio, judging by that the anticancer effect of combination treatment was more predominant in T47D cells than that in MCF-7 and MDA-MB-231 cells [60]. Additionally, in mouse mammary tumor virus erbB2 female transgenic mice administrated with different doses of 17β-oestradiol, it was found that the breast cancer incidence of mice was reduced by soybean diet in a high-oestrogen environment, but increased in a low-oestrogen environment [61].

Soy isoflavones also showed inhibitory effects on the ER-negative MDA-MB-231 breast cancer cells [62,63], indicating that apart from interacting with ER, soy isoflavones also exert anti-breast cancer effect through various ER-independent mechanisms. For instance, soy products and soy isoflavones could induce apoptosis in both ER+ and ER− breast cancer cells. A water-soluble extract of long-term fermented doenjang (a fermented soybean product in Korea) induced cell cycle arrest, proliferation inhibition, and consequential apoptosis in breast cancer cells [64]. In addition, growth inhibition and apoptosis were induced by genistein in MCF-7-C3 and T47D cells, through downregulation of the cancerous inhibitor of protein phosphatase 2A, an oncogene found overexpressed in breast cancer [65]. Genistein also induced apoptosis in MCF-7 cells via the inactivation of the IGF-1R/p-Akt signaling pathway and decreasing the Bcl-2/Bax mRNA and protein expression [66]. Besides, 6,7,4′-trihydroxyisoflavone, a metabolite of daidzein, induced apoptosis in MCF10CA1a cells, through upregulation of DR4 expression and downregulation of XIAP, leading to the PARP cleavage. This metabolite also induced cell cycle arrest at S- and G_2_/M phases by regulating cyclins and cyclin-dependent kinases (CDKs) [67]. Besides, extract of a soybean biotransformed by fungus induced cell death on MCF-7 cells, which was associated with the activation of caspase-3 and upregulation of proapoptotic molecule expression [68].

Soy isoflavones could also cause epigenetic alterations in breast cancer cells. Genistein inhibited DNA methylation and increased expression of some tumor suppressor genes in breast cancer cells, which might partly contribute to the anticancer effect of genistein [69]. In addition, quantitative phosphoproteomics revealed that genistein inhibited the growth of TNBC cell through modulating the DNA damage response and cell cycle in a more complex manner [70]. Furthermore, several prosurvival signalings in breast cancer cells were blocked by soy isoflavones. Genistein dose-dependently inhibited the growth of MDA-MB-231 cells by inhibiting activity of NF-κB via the Nocth-1 signaling pathway [71]. Genistein also decreased the breast cancer stem-like cell population both in vitro and in vivo, through downregulation of the Hedgehog-Gli1 signaling pathway [72]. Equol is a bacterial metabolite of daidzein. A study found that daidzein, *R*-(+)equol and *S*-(−)equol showed inhibition on the invasion of MDA-MB-231 cells through the downregulation of MMP-2 expression [63].

The controversy also exists in the results of experimental studies. Several recent studies challenged the inhibitory effect of soy isoflavones on breast cancer. For instance, a study found that isoflavone extracts of 51 commercial soybean cultivars were estrogenic and stimulated the growth of ER+ MCF-7 cells by 1.14 to 4.59 folds [73]. In addition, a study found that genistein at physiological concentration (5 µM) increased cellular levels of ROS and stimulated proliferation of breast cancer cell through the induction of CYP1B1 gene expression [74]. Moreover, long-term genistein treatment at low doses (≤500 ppm) promoted MCF-7 tumor growth and led to a more aggressive and advanced tumor growth phenotype after stimulus withdrawal [75]. Besides, in an experimental model of breast cancer with bone micro-tumors, soy isoflavones supplement (750 mg/kg) induced an increase of metastasis to lungs [76]. Furthermore, daidzein promoted growth of breast cancer cells in vitro (1 µg/mL) and stimulated estrogen-induced cell proliferation in rat uterus (0.066 mg/kg body weight (bw)), which suggested a caution for the use of daidzein in hormone replacement therapy [77]. Likewise, daidzein exposure promoted the expression of proto-oncogene BRF2 in ER+ breast cancer cells, through enhancing the demethylation and/or mRNA stabilization [78]. In addition, overexpression of ABC drug transporters is a contributor to multidrug resistance. Genistein treatment increased the expression of ABCC1 and ABCG2 at the protein level in MCF-7 cells, leading to an enhancement in doxorubicin and mitoxantrone efflux and resistance [79]. 

Collectively, most studies have supported a protective role of soy isoflavones against breast cancer, though some adverse effects have also been reported. Nevertheless, it should be noted that since the intrinsic cellular pathways often interfere or overlap, there was not a clear boundary between ER-dependent and ER-independent mechanisms under the anti-breast cancer action of soy isoflavones. Meanwhile, whether soy isoflavones promote or inhibit the growth of breast cancer seemed to be dependent on their doses [80], thus the dosage and long-term safety of soy isoflavones need to be further investigated before they are recommended as supplement for breast cancer patients.

## 3. Fruits

Fruits normally contain high content of polyphenols, which gives fruits great antioxidant activity and may help reduce risk of cancer [81,82,83,84,85]. In a meta-analysis which included fifteen prospective studies, high intake of fruits was associated with a weak reduction in risk of breast cancer (summary RR for the highest versus the lowest intake was 0.92, 95% CI = 0.86–0.98, *I*^2^ = 9%) [86]. Besides, another meta-analysis suggested a borderline inverse association between pre-diagnostic fruit intake and the overall survival of breast cancer (summary HR for the highest versus the lowest intake was 0.83, 95% CI = 0.67–1.02, *I*^2^ = 0%) [87]. Some fruits, such as pomegranate, mangosteen and citrus fruits, have shown inhibition on breast cancer cells.

### 3.1. Pomegranate

Pomegranate (*Punica granatum* L.) has been utilized for medicinal purposes for centuries and is described as “nature’s power fruit” [88]. Pomegranate fruit contained a high content of polyphenols, among which ellagitannins predominate, and showed great antioxidant activity and anti-inflammatory properties [89,90]. Pomegranate extract (PE) inhibited MCF-7 breast cancer cell growth by inducing cell cycle arrest in G_2_/M phase and inducing apoptosis, and the effects might be associated with downregulation of homologous recombination, which could sensitize cancer cells to double strand breaks [91]. Another study revealed that PE exerted proapoptotic and antiproliferative effects on DMBA-inflicted rat mammary tumorigenesis, possibly through concurrent disruption of ER and Wnt/-catenin signaling pathways [92]. Furthermore, hydrophilic fraction of pomegranate seed oil significantly decreased cell viability of MCF-7 and MDA-MB-231 breast cancer cell lines and induced cell cycle arrest in G_0_/G_1_ phase [93]. Besides, pomegranate juice or combined with its components (luteolin + ellagic acid + punicic acid) could block the metastatic processes of breast cancer cells, as shown by inhibited cell growth, increased cell adhesion and decreased cell migration [94]. Another study indicated that PE showed anticancer activities on breast cancer cells, which was partly due to targeting microRNAs155 and 27a [95]. Additionally, pomegranate peel extract reduced cell proliferation and induced apoptosis on MCF-7 cancer cells, by increasing expression of Bax and decreasing the expression Bcl-2 [96]. Another study found that a pomegranate extract consisting of fermented juice and seed oil could inhibit invasion and motility of human breast cancer by inhibiting RhoC and RhoA protein expression. The bioactive components were identified as ellagitannins and phenolic acids in the aqueous extract, and conjugated octadecatrienoic acids in the lipid extract of seed [97]. Besides, pomegranate ellagitannin-derived compounds inhibited aromatase activity and proliferation of breast cancer cell line, indicating a potential for the prevention of estrogen-responsive breast cancers [98]. Furthermore, the whole pomegranate seed oil and fermented pomegranate juice polyphenols both inhibited the cancerous lesion formation induced by DMBA in a murine mammary gland organ culture, suggesting a chemopreventive property and adjuvant therapeutic potential of pomegranate [99,100].

### 3.2. Mangosteen

Mangosteen (*Garcinia mangostana* L.) known as “queen of fruits” is a common tropical fruit. Crude methanolic extract of mangosteen pericarp could inhibit proliferation and induce apoptosis on SKBR3 human breast cancer cell line [101]. In addition, phenolics from mangosteen fruit pericarp produced great cytotoxicities against MCF-7 human breast cancer cells [102].

Mangosteen pericarp is a rich source of xanthones, such as α- and γ-mangostin, which have a variety of bioactivities, such as antioxidant, anti-inflammatory, and anticancer activities [103]. In a study, twelve xanthone constituents were isolated from the pericarp of mangosteen, among which α-mangostin, γ-mangostin, garcinone D, and garcinone E, showed dose-dependent anti-aromatase activity in SK-BR-3 breast cancer cells, with γ-mangostin being the most potent [104]. Furthermore, a study showed that α-mangostin could induce apoptosis in T47D breast cancer cells through modulating HER2/PI3K/Akt and MAPK signaling pathways [105]. In addition, α-mangostin treatment on MDA-MB231 cell line carrying a p53 mutation induced mitochondria-mediated apoptosis and cell cycle alterations (G_1_-phase arrest, upregulation of p21(cip1) expression and downregulation of cyclins, cdc(s), CDKs and PCNA) [106]. Besides, α-mangostin isolated from mangosteen exerted cytotoxicity on SKBR3 breast cancer cells and showed apoptotic bodies [107]. In addition, α-mangostin effectively inhibited fatty acid synthase (FAS) expression and intracellular FAS activity, and induced apoptosis in human breast cancer cells [108].

Furthermore, a study revealed that the presence of ERα is necessary for growth inhibition and apoptosis induced by α-mangostin in human breast cancer cells, as evidenced by that MDA-MB-231 cells (ER-α negative) is less sensitive to α-mangostin than MCF-7 cells (ERα positive), and that knockdown of ERα reduced the cell growth inhibition and caspase-7 activation induced by α-mangostin [109]. Besides, lymph node metastasis partly contributes to the lethality of breast cancer. In a study, α-mangostin treatment (20 mg/kg/day) significantly increased survival rate of mice carrying mammary tumors, and greatly suppressed tumor volume and the multiplicity of lymph node metastases. In vitro studies showed that α-mangostin induced mitochondria-mediated apoptosis and G_1_- and S-phase cell cycle arrest, and decreased levels of phospho-Akt-threonine 308 (Thr308) [110]. Besides, treatment of panaxanthone (approximately 80% α-mangostin and 20% γ-mangostin) isolated from pericarp of mangosteen significantly suppressed mammary tumor volumes in mice, and decreased the multiplicity of lung metastasis and lymph node metastasis. These effects were associated with increases of apoptotic cell death, antiproliferation and antiangiogenesis. The in vitro analysis also confirmed that α-mangostin induced apoptosis on BJMC3879 cells [111].

### 3.3. Citrus Fruits

Citrus fruits include a large class of fruits, such as orange, lemon, grapefruit, pomelo and lime. Recently the anti-breast cancer activity of citrus fruits has attracted increasing attention. A meta-analysis of observational studies pointed out an inverse association between citrus fruits intake and the risk of breast cancer (OR, 0.90; 95% CI = 0.85–0.96; *p* < 0.001) [112].

Polysaccharides from Korean Citrus hallabong peels inhibited angiogenesis as shown by reducing tube formation of human umbilical vein vascular endothelial cells, and suppressed cell migration of MDA-MB-231 cells via downregulation of MMP-9 [113]. Besides, extracts from a citrus fruit named Phalsak induced apoptosis in anoikis-resistant breast cancer stem cell line MCF-7-SC [114]. In addition, lemon citrus extract induced apoptosis in MCF-7 breast cancer cells via upregulating the expression of bax and caspase-3 genes, and downregulating the expression of bcl-2 gene [115]. Furthermore, naringin, a flavonoid presenting abundantly in citrus fruits, inhibited cell proliferation, and promote cell apoptosis and G_1_ cycle arrest in TNBC cell lines-based in vitro and in vivo models through modulating β-catenin pathway [116]. Besides, hesperidin, a flavonoid derived from citrus fruits, showed inhibitory effect on the proliferation of MCF-7-GFP-Tubulin cells [117].

### 3.4. Apple

Apple is widely consumed and an important part of the human diet. Flavonoids extracted from the peel and flesh of Pink Lady apples could both inhibit MCF-7 breast cancer cell growth, with IC_50_ of 58.42 ± 1.39 mg/mL and 296.06 ± 3.71 mg/mL, respectively [118]. Besides, another study investigated an apple cultivar called Pelingo, and found that the Pelingo apple juice contained high content of polyphenol and exerted antiproliferative effect on MCF-7 and MDA-MB-231 cells. Pelingo juice also inhibited 12-o-tetra-decanoyl-phorbol-13-acetate (TPA)-induced tumorigenesis of pre-neoplastic cells, by inhibiting colony formation and TPA-induced ERK1/2 phosphorylation [119]. Additionally, apple extract showed a significant antiproliferative effect on MCF-7 and MDA-MB-231 cells at concentrations of 10–80 mg/mL (*p* < 0.05). Apple extract also significantly induced cell cycle arrest at G_1_ phase in MCF-7 cells by decreasing cyclin D1 and Cdk4 proteins [120]. Another study showed that apple extract and 2α-hydroxyursolic acid isolated from apple peel could both inhibit NF-κB activation induced by TNF-α in MCF-7 cells through suppressing the proteasomal activities [121,122]. In addition, 2α-hydroxyursolic also showed antiproliferative and pro-apoptotic effects on MDA-MB-231 cells by regulating the p38/MAPK signal transduction pathway [123]. Furthermore, pectic acid isolated from apple could induce apoptosis and inhibit cell growth of 4T1 breast cancer cells in vitro, and prevent tumor metastasis in BALB/c mice via overexpression of p53 [124]. Results of another study indicated a synergistic inhibitory effect of the quercetin 3β-d-glucoside and apple extract combination on the proliferation of MCF-7 cells [125].

### 3.5. Grape

Grape and its products such as wine are well recognized healthy food consumed in the world. Dietary grape skin extract (0.5 and 1.0 mg/mL in drinking water) significantly inhibited the lung metastasis of breast tumor in Balb/c mice implanted with 4T1 cells. In vitro study revealed that grape skin polyphenols inhibited migration of 4T1 cells, which might be associated with blocking the PI3k/Akt and MAPK pathways [126]. Another study showed that grape seed extract suppressed migration and invasion of the highly metastatic MDA-MB231 cells, possibly through inhibiting β-catenin expression and localization, decreasing fascin and NF-κB expression and the activities of urokinase-type plasminogen activator (uPA), MMP-2 and MMP-9 [127]. In addition, a red grape wine polyphenol fraction showed selective cytotoxicity on MCF-7 cells, as evidenced by membrane damage, disrupted mitochondrial function and G_2_/M cell cycle arrest [128]. Another study screened *Vitis amurensis* grape for active compounds to inhibit vascular endothelial growth factor (VEGF) production in tamoxifen-resistant MCF-7 cells, and amurensin G presented to be the most potent one. The effect was through blocking Pin1-mediated VEGF gene transcription [129]. Furthermore, muscadine grape skin extract could decrease cell invasion, migration and bone turnover in MCF-7 cells, via inhibiting expression of Snail and phosphorylated signal transducers and activators of transcription 3 (STAT3) and abrogating Snail-mediated CatL activity [130].

### 3.6. Mango

Mango (*Mangifera indica* L.) is a commonly cultivated tropical fruit and rich in polyphenolic compounds such as gallic acid and gallotannins. A study found that mango polyphenolics exhibited cytotoxic effects on BT474 cells in vitro, and decreased tumor volume by 73% in mice bearing BT474 xenograft compared with control group. These effects were partially regulated through the PI3K/AKT pathway and miR-126 [131]. Another study investigated three genetically diverse mango varieties, and found that the peel extract of Nam Doc Mai mango contained the highest amounts of polyphenols, inhibited cell viability of MCF-7 cells with an IC_50_ of 56 µg/mL, and significantly (*p* < 0.01) stimulated cell death in MDA-MB-231 cells [132]. Furthermore, ethanolic extract of mango seed induced apoptosis in MCF-7 and MDA-MB-231 cells, through increasing pro-apoptotic proteins (cytochrome c, Bax, caspase-7, -8 and -9) and decreasing anti-apoptotic proteins (p53, Bcl-2, and glutathione). The effects were also associated with the activation of oxidative stress in breast cancer cells [133,134]. Besides, gallotannins in mango are generally not absorbable, therefore they are rarely studied for bioactivities. A study found that pyrogallol, the major microbial metabolite of gallotannins, and a mango polyphenols fraction could both inhibit breast cancer ductal carcinoma in situ proliferation in vitro, which was possibly through mediating the AKT/mTOR signaling pathway [135].

### 3.7. Other Fruits

Jujube (*Ziziphus jujube*) fruit has shown numerous medicinal and pharmacological effects, such as antioxidant and anti-inflammatory activities [136]. A study found that *Ziziphus jujube* extracts induced cell death by apoptosis in MCF-7 and SKBR3 breast cancer cells, without decreasing cell viability of nonmalignant breast epithelial MCF-10A cells or normal human fibroblasts BJ1-hTERT [137]. In addition, betulinic acid was isolated from sour jujube fruit, and microencapsulated betulinic acid could induce apoptosis in MCF-7 cells, through the mitochondria transduction pathway [138]. Furthermore, jujube aqueous extract treatment on MCF-7 cells exhibited antiproliferative and pro-apoptotic effects, through upregulating expression of Bax and downregulating Bcl2 gene [139].

Some berry fruits except for grape mentioned above also showed inhibitory effects on breast cancer cells. Methanolic extract of strawberry exerted cytotoxicity in T47D breast cancer cells in vitro, and inhibited the proliferation of tumor cells in mice bearing breast adenocarcinoma by activating apoptosis [140]. Besides, bilberry extract inhibited proliferation of MCF-7 cells in a concentration-dependent manner (IC_50_ = 0.3–0.4 mg/mL), accompanied by induction of apoptotic cell death [141]. Furthermore, Jamun is the ripe purple and edible berries of the plant *Eugenia jambolana* Lam, and widely consumed in the United States. In a study, Jamun fruit extract showed great antiproliferative and pro-apoptotic effect on MCF-7aro and MDA-MB-231 cells, but only mild antiproliferative activity and no pro-apoptotic effect on the normal MCF-10A cells [142]. Additionally, cranberry extract could inhibit the proliferation of MCF-7 cells, which was partly attributed to the induction of apoptosis and G_1_ phase arrest [143].

Polyphenolics from peach (*Prunus persica*) suppressed breast tumor growth and lung metastasis in a dose range of 0.8–1.6 mg/day in mice (about 370.6 mg/day for a human adult of 60 kg), which was regulated by inhibition of MMPs gene expression [144]. Another study investigated the anticancer effect of plums (*Prunus salicina*), and found that immature plums exhibited higher cytotoxic effects against MDA-MB-231 cells than mid-mature and mature plums, and contained higher levels of total phenolics and condensed tannins. The immature plums also induced apoptosis in MDA-MB-231 cells, associated with increased Bax levels, decreased Bcl-2 levels and the cleavage of caspases and PARP [145]. Besides, flavanols from Japanese quince (*Chaenomeles Japonica*) fruit exerted antiproliferative activity and inhibited invasiveness in MDA-MB-231 cells [146]. Additionally, a graviola fruit extract selectively inhibited the growth MDA-MB-468 breast cancer cells (IC_50_ = 4.8 µg/mL), without affecting nontumorigenic MCF-10A breast epithelial cells in vitro. Furthermore, 5-week dietary treatment of this extract (200 mg/kg) inhibited tumor growth by 32% (*p* < 0.01) in mouse xenograft model, through the EGFR/ERK signaling pathway [147]. In addition, litchi fruit pericarp extract inhibited cell growth (IC_50_ = 80 µg/mL) of human breast cancer cells dose- and time-dependently in vitro, and 0.3 mg/mL oral administration of the extract for 10 weeks reduced tumor mass volume by 40.70% in mice, through multiple mechanisms [148]. Besides, bromelain isolated from the stems and immature fruits of pineapple induced cell death of GI-101A breast cancer cells in vitro by promoting apoptosis [149].

Collectively, the intake of fruits is generally beneficial for the prevention and treatment of breast cancer, and pomegranate, mangosteen, apple, citrus fruits, grape and mango have shown the most promising effects. The anti-breast cancer action of these fruits might be attributed to the presence of some bioactive component, such as ellagitannins in pomegranate and mangostin in mangosteen.

## 4. Vegetables

A meta-analysis of prospective studies indicated that high intake of fruits and vegetables combined was associated with a weak reduction in risk of breast cancer, with the summary relative risk (RR) for the highest versus the lowest intake of 0.89 (95% CI: 0.80–0.99, *I*^2^ = 0%). However, no significant association was found between vegetable intake alone and risk of breast cancer [86]. In experimental studies, several vegetables, especially cruciferous vegetables, have shown inhibitory effect on breast cancer cells.

### 4.1. Cruciferous Vegetables

Cruciferous vegetables, such as broccoli, cauliflower, watercress and Brussel sprouts, are grown and consumed worldwide. According to a meta-analysis with 13 epidemiologic studies included, intake of cruciferous vegetables was inversely associated with risk of breast cancer (RR = 0.85, 95% CI = 0.77–0.94) [150]. Cruciferous vegetables have shown anti-breast cancer effect on experimental models, which might be attributed to its high contents of glucosinolates. When the vegetables are cut or chewed, the enzyme myrosinase is released, and glucosinolates would be degraded to form isothiocyanates. Isothiocyanates include a variety of compounds such as benzyl isothiocyanate, phenethyl isothiocyanate and sulforaphane, and have been long known to have chemopreventive activities for various neoplasms including breast cancer [151,152]. Besides, the indole-3-carbinol in cruciferous vegetables and its metabolite 3,3′-diindolylmethane also showed anti-breast cancer action [153,154].

#### 4.1.1. Isothiocyanates

Benzyl isothiocyanate (BITC)-induced inhibition on breast cancer cells is associated with apoptotic cell death, and inhibition of mitochondrial fusion was found to be an early and critical event involved in BITC-induced apoptosis [155]. Meanwhile, it was found that BITC-induced apoptosis in MCF-7 and MDA-MB-231 cells was not p53-dependent, but mediated by suppression of XIAP expression [156]. In addition, in breast cancer cells treated with BITC, the proapoptotic proteins Bax and Bak were upregulated and the antiapoptotic proteins Bcl-2 and Bcl-xL were downregulated, indicating that apoptosis was induced by BITC. Generation of ROS and cleavage of caspase-9, caspase-8, and caspase-3 were also involved in this process [157]. BITC could also inhibit the migration and metastasis of human breast cancer cells. On the one hand, BITC markedly suppressed the invasion and migration of MDA-MB-231 cells, which was involved with reduced uPA activity, and suppression of Akt signaling [158]. On the other hand, epithelial-mesenchymal transition (EMT) process was triggered during progression of cancer to invasive state [159]. BITC treatment inhibited TGF β-/TNF α-induced migration via suppression on EMT process, as shown by the upregulated epithelial markers (E-cadherin and occludin), and downregulated mesenchymal markers (vimentin, fibronectin, snail, and c-Met), both in vitro and in vivo [160]. Besides, BITC exposure caused FoxO1-mediated autophagic death in breast cancer cells and MDA-MB-231 xenografts [161]. BITC could also act against the oncogenic effects of leptin on MDA-MB-231 and MCF-7 cells through suppressing activation of signal transducer and activator of transcription 3 [162]. BITC inhibited the growth of MDA-MB-231 xenografts by suppression on cell proliferation and neovascularization [163]. Additionally, BITC treatment inhibited breast cancer stem cells in vitro and in vivo, possibly by targeting Ron receptor tyrosine kinase [164].

Phenethyl isothiocyanate (PEITC), another natural isothiocyanate, also showed growth inhibition on breast cancer cells. Pharmacological concentrations of PEITC induced a PUMA-independent apoptosis on BRI-JM04 breast cancer cells, which was mediated by Bim [165]. Besides, PEITC suppressed adhesion, aggregation, migration and invasion of MCF-7 and MDA-MB-231 cells via modulation of HIF-1α [166]. Moreover, PEITC administration significantly prolonged the tumor-free survival and reduced the tumor incidence induced by *N*-methyl nitrosourea (NMU) in rats, since the tumor incidences were 56.6%, 25.0% and 17.2% for control, 50 µmol/kg, and 150 µmol/kg group, respectively. This chemopreventive activity might be attributed to its anti-angiogenic effects [167]. Besides, PEITC treatment (3 µM) to MCF-7 cells caused alterations in some genes in breast cancer, such as p57 Kip2, p53, BRCA2, IL-2, and ATF-2, which were involved in tumor suppression and cellular proliferation/apoptosis [168].

Sulforaphane (SFN), is also a potent inhibitor of mammary carcinogenesis through various mechanisms of action. SFN could downregulate ERα expression in MCF-7 cells, partially by blocking ERα mRNA transcription and increasing proteasome-mediated degradation [169]. SFN also induced cell type-specific apoptosis in breast cancer cells, since SFN-activated apoptosis in different breast cancer cell lines was initiated through different signaling pathways. To be specific, SFN activated apoptosis in MDA-MB-231 cells through induction of Fas ligand which led to activation of caspase-8, caspase-3 and poly (ADPribose) polymerase, while SFN induced apoptosis in the other breast cancer cell lines by reduction of Bcl-2 expression, release of cytochrome c into the cytosol, activation of caspase-3 and caspase-9, but not caspase-8, and poly (ADP-ribose) polymerase cleavage [170]. In addition, SFN suppressed the growth of KPL-1 human breast cancer cells both in vitro and in athymic mice [171], and the anti-metastatic action of SFN might be through inhibiting MMP-9 expression via the NF-κB signaling pathway [172]. Moreover, it was found that SFN showed antiproliferative effect on various TNBC cells through activating tumor suppressor Egr1 [173]. Besides, SFN also downregulated telomerase in breast cancer cells by inducing epigenetic repression of hTERT expression [174]. In addition, a study found that SFN showed anticancer efficacy in ER+ and COX-2 expressed breast cancer, which might be mediated by p38 MAP kinase and caspase-7 activations [175].

#### 4.1.2. Indole-3-Carbinol

Indole-3-carbinol (I3C) is found at high concentrations in Brassica vegetables, and is a natural anti-carcinogenic compound. Studies have indicated that I3C showed anti-breast cancer action, since it could interact directly with the ERα and inhibit its activity, or through estrogen-independent actions, such as blocking cell cycle progression and metastasis, and inducing apoptosis. I3C (50 or 100 µM) could suppress the cell adhesion, migration, and invasion in vitro as well as the in vivo lung metastasis formation in MCF-7 and MDA-MB-468 cell lines, which was associated with upregulation of BRCA1 and E-cadherin/catenin complexes [176]. In addition, I3C pretreatment inhibited the migration through suppressing the EMT process and downregulating FAK expression [177]. I3C-induced inhibition on MMP-2 by blocking the ERK/Sp1-mediated gene transcription also contributed to its anti-invasive action on breast cancer cells [178]. Furthermore, I3C could induce inhibition on breast cancer bone metastasis by inhibiting CXCR4 and MMP-9 expression through downregulation of the NF-κB signaling pathway [179]. I3C could also regulate the cell cycle progression of breast cancer cells. For instance, I3C could inhibit CDK2 function in MCF-7 cells, by regulating cyclin E composition, the size distribution, and subcellular localization of the CDK2 protein complex [180]. Moreover, I3C suppressed CDK6 expression in MCF-7 cells, via targeting Sp1 at a composite DNA site in the CDK6 promoter [181]. Moreover, I3C downregulated expression of telomerase gene through disruption of the combined ERα- and Sp1-driven transcription of hTERT gene expression, leading to a cell cycle arrest in breast cancer cell [182]. Besides, I3C could induce apoptotic cell death in MDA-MB-435 and MCF10CA1a breast cancer cells, mainly through inducing overexpression and translocation of Bax to mitochondria, resulting in mitochondrial depolarization and activation of caspases [183,184]. In addition, I3C exerted antiproliferative action on estrogen-sensitive MCF-7 breast cancer cells, via suppressing the expression of IGF1R and IRS1, which was dependent on downregulation of ERα [185]. Furthermore, I3C induced stress fibers and focal adhesion formation through upregulation of Rho kinase activity, leading to an inhibited motility of MDA-MB-231 cell [186]. Interferon gamma (IFNγ) played a key role in prevention of the development of primary and transplanted tumors [187]. The anti-breast cancer effect of I3C might also be through stimulating expression of interferon gamma receptor 1 (IFNγR1) and augmenting the IFNγ response [188].

#### 4.1.3. 3,3′-Diindolylmethane 

**Figure 3:**
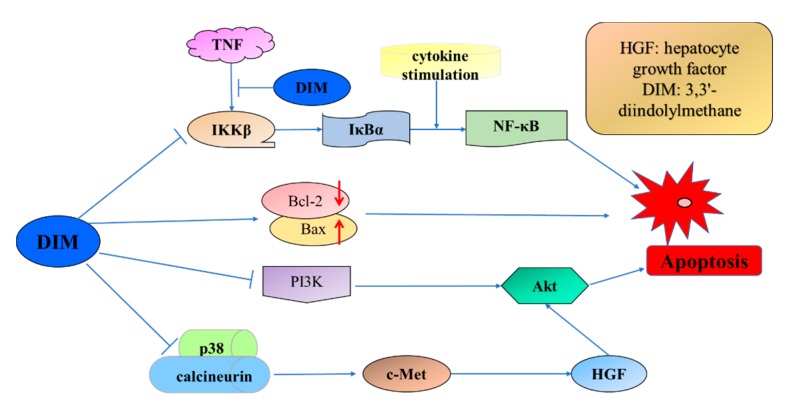
Signaling pathways involved in DIM-induced apoptosis in breast cancer cells.

3,3′-Diindolylmethane (DIM) is an in vivo acid-catalyzed condensation product of I3C and much more stable than I3C. DIM is also a promising anticancer agent. Multiple targets and underlying mechanisms of DIM-induced inhibition on breast cancer cells have been found. For instance, DIM induced apoptosis in MCF-7 and MDA-MB-231 breast cancer cells (Figure 3), by decreasing total transcript and protein levels of Bcl-2 and increasing Bax protein levels [189]. Apoptosis induced by DIM in MCF10CA1a breast cancer cells was also modulated by inactivation of Akt and NF-κB [190]. Another study revealed the upstream mechanism of DIM-induced inhibition on Akt in MDA-MB-231 breast cancer cells, which was through blockade of hepatocyte growth factor/c-Met signaling [191]. Meanwhile, DIM inhibited breast cancer cell proliferation and induced cell cycle arrest in G_2_/M phase in MCF-7 breast cancer cells, via enhancing miR-21-mediated Cdc25A degradation [192]. Meanwhile, DIM treatment markedly increased the portion of cells in G_1_ phase through Sp1/Sp3-induced activation of p21 expression [193]. The upstream events leading to DIM-induced p21 overexpression were further studied, and results showed that DIM could act as a strong mitochondrial H^+^-ATP synthase inhibitor. Hyperpolarization of mitochondrial inner membrane was induced by DIM treatment, which decreased cellular ATP level and markedly promoted mitochondrial ROS production, and in turn induced p21^Cip1/Waf1^ expression [194]. Besides, DIM lowered the invasive and metastatic potential of breast cancer cells through downregulation of CXCR4 and CXCL12 [195]. In addition, survivin was found to be another target of cell growth inhibition and apoptosis induced by DIM in MDA-MB-231 breast cancer cells [196]. Like I3C, DIM could also stimulate the expression and secretion of IFNγ in MCF-7 cells through the activation of JNK and p38 pathways [197].

### 4.2. Other Vegetables

It is reported that the extract of red beetroot (*Beta vulgaris* L.) exhibited a dose-dependent cytotoxic effect on MCF-7 cells in vitro [198]. Besides, a randomized study pointed out that daily intake of 8 ounces of fresh Balero or BetaSweet orange carrot juice increased plasma level of total carotenoid by 1.65 and 1.38 µM respectively in overweight breast cancer survivors. This increase in total plasma carotenoids was inversely associated with the level of 8-iso-PGFα, which was used as an oxidative stress marker (OR: 0.13; 95% CI = 0.20–0.75). These results indicated that daily intake of fresh carrot juice might benefit patients with breast cancer [199].

Collectively, cruciferous vegetables have shown a potential role in the prevention and treatment of breast cancer, and the main bioactive components are isothiocyanates (including BITC, PEITC, SFN), indole-3-carbinol and its metabolite 3,3′-diindolylmethane. The underlying mechanisms mainly include down-regulating ERα and repressing ER signaling, inducing apoptosis and cell cycle arrest, and inhibiting the metastasis of breast cancer cells.

## 5. Spices

Spices have been widely used in folk medicines and as food flavorings for a long time. In recent years, several spices and their bioactive constituents, such as gingerols and shogaols in ginger, organosulfur components in garlic, and thymoquinone in black cumin, have been suggested to possess anti-breast cancer activity.

### 5.1. Ginger

Ginger (*Zingiber officinale*) is a commonly used spice around the world for dietary and medicinal purpose since ancient period. Ginger has shown anti-breast cancer effect in recent researches. For instance, methanolic extract of ginger exhibited inhibitory effect on the proliferation and colony formation in MDA-MB-231 cells dose- and time-dependently [200]. Ginger extract also induced apoptosis in MCF-7 and MDA-MB-231 cells, via up-regulation of Bax, and downregulation of Bcl-2 proteins, NF-κB, Bcl-X, Mcl-1, survivin, cyclin D1 and CDK-4. Besides, the expression of c-Myc and hTERT, the two prominent molecular targets of cancer, was inhibited by ginger extract [201]. The anti-breast cancer property of ginger might be attributed to the bioactive constituents in ginger, such as gingerols and shogaols.

Gingerols showed inhibition on the proliferation and metastasis of breast cancer cells [202,203]. 10-Gingerol inhibited proliferation of MDA-MB-231 through inhibition on cyclin-dependent kinases and cyclins, leading to a G_1_ phase arrest. Invasion of cancer cell was also inhibited by 10-gingerol through suppression of Akt and p38 (MAPK) activity [202]. Besides, 6-gingerol exerted a concentration-dependent inhibition on migration and motility of MDA-MB-231 cells, accompanied with a decreased of expression and activities of MMP-2 and -9 [203].

Shogaols also inhibited metastasis of breast cancer cells through different mechanisms [204,205]. 6-Shogaol reduced expression of MMP-9 via blockade of NF-κB activation, leading to an inhibited invasion of MDA-MB-231 cells [204]. Besides, 6-shogaol inhibited invadopodium formation by decreasing levels of c-Src kinase, cortactin, and MT1-MMP, which are key modulators of invadopodium maturation, thereby inhibiting invasion of MDA-MB-231 cells [205]. Besides, 6-shogaol could inhibit the growth and sustainability of spheroid generated from adherent breast cancer cells. This effect was through γ-secretase-mediated downregulation of Notch signaling and induction of autophagic cell death [206]. 6-Dehydrogingerdione is also an active constituent of dietary ginger, and induced apoptosis and cell cycle arrest in G_2_/M phase in MCF-7 and MDA-MB-231 cells through mediation of ROS/JNK pathways [207].

Furthermore, several clinical studies suggested a beneficial effect of ginger on patients with breast cancer. In patients receiving oral supplement of ginger, nausea severity and the number of vomiting episodes were significantly reduced than those of control group [208]. Besides, a single-blind, controlled, randomized cross-over study revealed that breast cancer patients who received ginger essential oil inhalation showed significantly lower nausea scores during acute phase, but no significant difference in overall treatment effect. Besides, ginger aromatherapy improved the baseline for global health status and appetite loss, while the vomiting was not improved [209].

### 5.2. Garlic

Garlic (*Allium sativum*) as a spice has been used worldwide. Meanwhile, it has been used in folk medicine to treat a variety of ailments [210]. A recent case-control study suggested that high consumption of certain *Allium* vegetables, especially garlic, is associated with a decreases risk of breast cancer, with adjusted ORs of 0.41 (95% CI = 0.20–0.83) [211]. Experimental studies indicated that the anti-breast cancer property of garlic might be attributed to organosulfur components, including diallyl disulfide [212], diallyl trisulfide [213], *S*-allyl mercaptocysteine [214], and allicin [215].

Diallyl disulfide (DADS) is one of the major organosulfur compounds isolated from garlic oil, and could induce apoptosis in MCF-7 breast cancer cells [216,217]. The pro-apoptotic effect might be through inhibition of histone deacetylation [216] and inhibition of ERK and the activation of the SAPK/JNK and p38 pathways [217]. Besides, DADS inhibited proliferation and metastasis of human breast cancer [218,219]. The DADS treatment upregulated expression of miR-34a, which led to inhibition on SRC expression and consequently triggered the blockade of the SRC/Ras/ERK pathway, ultimately led to an inhibitory effect on the proliferation and metastasis of MDA-MB-231 cells [218]. The DADS treatment also inhibited growth and metastatic potential of TNBC cells through inactivation of the β-catenin signaling pathway [219].

Diallyl trisulfide (DATS) also exhibited inhibitory effect on breast cancer by inducing apoptotic cell death [220,221]. It induced apoptosis in both MCF-7 cells and tumor xenografts by overproduction of ROS and subsequent activation of JNK and AP-1 [220]. The DATS also induced apoptosis in MCF-7 cells through upregulating the expression level of FAS, cyclin B1, cyclin D1, Bax and p53, and downregulating expression of Akt and Bcl-2 [221]. Furthermore, DATS inhibited migration and invasion of TNBC cells, through inhibiting MMP2/9 by suppressing NF-κB and ERK/MAPK signaling pathways [222]. Besides, a study indicated ERα might be a target of DATS in breast cancer cells, since DATS inhibited the expression and activity of ERα in MCF-7 and T47D cells. Peptidyl-prolyl *cis-trans* isomerase (Pin1) partially accounted for ERα protein suppression induced DATS treatment in MCF-7 cells [213]. Forkhead Box Q1 (FoxQ1) might be another novel target of DATS in breast cancer stem cell [223]. Pharmacological concentrations of DATS (2.5 and 5 µM) induced a dose-dependent inhibition on MCF-7 and SUM159 cells, which was associated with a decreased protein level of FoxQ1 [223].

S-allyl mercaptocysteine (SAMC), a water-soluble constituent derived from garlic, effectively inhibited cell growth of MCF-7 and MDA-MB-231 cells, by inducing apoptosis and cell cycle arrest in G_0_/G_1_ phase [214]. The mitochondrial apoptotic pathway was triggered by SAMC treatment, as shown by upregulation of Bax, downregulation of Bcl-2 and Bcl-X-L, and activation of caspase-9 and caspase-3 [214]. Another major component of garlic, allicin, inhibited the invasion and metastasis of MCF-7 cells induced by TNF-α, but not in MDA-MB-231 cells. The underlying mechanism was through suppressing the VCAM-1 through inhibiting ERK1/2 and NF-κB signaling pathways and increasing interaction between ERα and p65 [215].

### 5.3. Black Cumin

Black cumin (*Nigella sativa*) is a popular spice and has been used in folk medicine for over 1400 years. Recently, the anticancer effect of black cumin has attracted increasing attention. In a study, a supercritical CO_2_ extract of black cumin exhibited pro-apoptotic and anti-metastatic effect on MCF-7 cells in vitro [224]. Another study pointed out that the antiproliferative (IC_50_ = 62.8 µL/mL) and pro-apoptotic effects of black cumin extract were through mediating both the p53 and caspase pathways [225]. 

Thymoquinone (TQ) is the major bioactive component isolated from the seeds of *Nigella sativa*, and has shown potent chemopreventive and chemotherapeutic activities [226]. Firstly, studies indicated that TQ might be an Akt suppressor. Akt could be activated (phosphorylated) by PI3K, and promote cell survival by inhibiting apoptosis through inactivating downstream targets, such as Bcl-2 family member BAD and GSK-3β [227]. TQ induced cell cycle arrest and apoptosis in doxorubicin-resistant MCF-7 cells [228], and in T-47D and MDA-MB-468 cells [229], all by inhibiting Akt phosphorylation. TQ also inhibited tumor growth and induced apoptosis in mice bearing breast cancer xenograft, which might be through inducing p38 phosphorylation via ROS generation [230]. Besides, studies indicated that the antiproliferative effect of TQ on breast cancer might be through modulation of the PPAR-γ activation pathway [231], and by mediating expression of COX-2 and production of prostaglandin E2 through PI3K/p38 kinase pathway [232].

### 5.4. Other Spices

Red chili peppers of the genus *Capsicum* are popular spice worldwide, and contained certain amount of capsaicin (8-methyl-*N*-vanillyl-6-nonenamide), which has shown antiproliferative effect on breast cancer cells [233,234,235]. Capsaicin treatment for 24 h induced apoptosis in MCF-7 cells dose-dependently in vitro through a caspase-independent pathway [233]. Besides, capsaicin induced apoptosis in MCF-7 breast cancer cell, which was associated with inducing mitochondrial dysfunction [234]. In another study, capsaicin inhibited growth and blocked migration of MCF-7, MDA-MB231, T47D, SKBR-3 and BT-474 cell lines in vitro. In vivo, capsaicin decreased the volume of breast tumors in mice by 50% without noticeable drug side effects, and suppressed the progression of preneoplastic breast lesions by 80%. Mechanistically, these effects were through mediating the EGFR/HER-2 pathway [235]. However, it should be noted that the role of capsaicin in cancer is controversial. Some studies have indicated that capsaicin itself was mutagenic and promote tumor formation, and might increase the cancer risk in humans [236,237,238].

Piperine is an alkaloid isolated from black pepper (*Piper nigrum*), and has been reported to have anti-breast cancer activities. In a study, piperine exhibited growth, motility and metastasis inhibitory effects on TNBC cells in vitro, and suppressed the growth of TNBC xenografts in immune-deficient mice [239]. Another study found piperine induced cytotoxicity and apoptosis, and inhibited migration of HER2-overexpressing breast cancer cells in vitro. Piperine significantly inhibited HER2 and FAS expression, and downregulated EGF-induced MMP-9 expression via inactivation of AP-1 and NF-κB through modulating ERK1/2, p38 MAPK and Akt signaling pathways [240]. Moreover, piperine inhibited the growth of 4T1 cells (at doses of 35–280 pmol/L) time- and dose-dependently, and induced apoptosis (at doses of 70–280 pmol/L) in a dose-dependent manner, accompanying activation of caspase 3. Besides, injection of 5 mg/kg piperine dose-dependently inhibited the 4T1 tumor growth and significantly suppressed the lung metastasis in vivo [241]. 

Saffron (*Crocus sativus*), a well-known spice, is widely used in the Mediterranean, Indian and Chinese diet [242]. Saffron has showed anticancer effect in several studies, which was attributed to its bioactive compounds, such as crocin and crocetin [243,244]. According to an in vitro study, incubating the highly invasive MDA-MB-231 cells with crocetin (1 and 10 µM) significantly inhibited proliferation and invasion of cancer cells, and the effect was through downregulation of MMP expression [243]. Besides, a study found that crocin and crocetin both inhibited the incidence of *N*-methyl-*N*-nitrosourea (NMU)-induced breast tumors in rats. Moreover, crocetin was found to be a more effective chemopreventive agent than crocin at both the initiation and promotion stages [244]. Clove (*Syzygium aromaticum*) is commonly used as a spice and traditional Chinese medicine [245]. Eugenol is the major contributor to the bioactivities of clove. A study indicated that eugenol treatment inhibited the growth and proliferation of MCF-7 cells and induced apoptosis in vitro. Besides, the level of intracellular glutathione was decreased and the level of lipid peroxidation was elevated by eugenol treatment [246]. In another study, eugenol (2 µM) showed antiproliferative and proapoptotic activity both in vitro and in xenografted human breast tumors, which was mediated through targeting the E2F1/survivin pathway [247]. Besides, a supercritical fluid extract of rosemary (*Rosmarinus officinalis*) exhibited inhibitory effect on breast cancer cells through mediation of ERα and HER2 signaling pathways [248]. Wasabi (*Wasabia japonica*) is a popular spice in Japan. In a study, 6-(methylsulfinyl)hexyl isothiocyanate derived from wasabi exhibited proapoptotic effect on mice inoculated with MDA-MB-231 cells by inhibiting NF-κB and thus regulating the PI3K/AKT pathway [249]. Besides, coriander, a common culinary spice, has been reported for its health promoting effects. The coriander root extract exerted cytotoxicity on MCF-7 cells by affecting antioxidant enzymes, inducing G_2_/M phase arrest and apoptotic cell death, which was associated with death receptor and mitochondrial apoptotic pathways [250]. It should be noted that the turmeric and its main bioactive component curcumin are not discussed in this section because their effects on breast cancer have been extensively reviewed [251,252,253,254].

Collectively, ginger, garlic and black cumin have shown the most promising anti-breast cancer effects among various spices. More attention has been paid to the effects of bioactive components in spices, such as gingerols and shogaols in ginger, diallyl disulfide and diallyl trisulfide in garlic. However, some adverse results have also been reported, such as the cancer-promoting effect of capsaicin isolated from red chili peppers.

## 6. Edible Macro-Fungi

Several kinds of edible macro-fungi have shown inhibitory effect on breast cancer, such as *Antrodia camphorate*, oyster mushroom (*Pleutorus eous*), and lingzhi mushroom (*Ganoderma lucidum*). In a case-control study conducted among Korean women, a significant inverse association between mushroom consumption and breast cancer incidence was found in postmenopausal women (OR = 0.17, 95% CI = 0.05–0.54, trend *p* = 0.0037 for average frequency; OR = 0.16, 95% CI = 0.04–0.54, trend *p* = 0.0058 for daily intake). No significant association was found in premenopausal women [255]. Besides, according to a meta-analysis with 7 observational studies included, mushroom intake might be inversely associated with risk of breast cancer (RR = 0.94, 95% CI = 0.91–0.97 for postmenopausal women; RR = 0.96, 95% CI = 0.91–1.00 for premenopausal women) [256].

*Antrodia camphorate* is a medicinal mushroom widely used in Taiwan [257]. Methyl antcinate A (MAA) is an ergostane-type triterpenoid isolated from the fruiting bodies of *A. camphorate*. MAA suppressed the population of cancer stem-like cells in MCF-7 cell line through inhibiting Hsp27 expression and increasing expression of p53 and IκBα [257]. Besides, a polysaccharide (SP1) with a molecular weight of 56 kDa was isolated from the fruiting body of mushroom Huaier (*Trametes robiniophila* Murr*.*). SP1 induced apoptosis in MCF-7 cells through downregulation of metadherin, which was overexpressed in most cancers [258]. Furthermore, an acidic polysaccharide, isolated from *Pleurotus abalonus* fruiting body, showed antiproliferative and proapoptotic effect on MCF-7 cells via ROS-mediated mitochondrial apoptotic pathway [259]. Treatment of this polysaccharide caused reduction in mitochondrial membrane potential, activation of caspase-9/3, increase of Bax/Bcl-2 ratio, overproduction of intracellular ROS, and degradation of PARP [259]. In addition, spores and unpurified fruiting body of *Ganoderma lucidum* inhibited invasion of MDA-MB-231 cells via inhibiting the expression of uPA and uPA receptor as well as the secretion of uPA [260]. The crude extract of *Ganoderma lucidum* fruiting body also caused both apoptosis and necrosis in estrogen-independent cell line, MDA-MB-435 [261]. Additionally, the aqueous extract of white button mushroom (*Agaricus bisporus*) dose-dependently suppressed the aromatase activity in MCF-7aro cells, which is an aromatase-transfected breast cancer cell line [262]. Besides, polysaccharides (50–250 µg/mL) isolated from oyster mushroom (*Pleutorus eous*) suppressed angiogenesis by downregulating VEGF, and induced apoptosis in MCF-7 cells through ROS-dependent JNK activation and mitochondrial mediated mechanisms [263].

Collectively, the anti-breast cancer effects of edible macro-fungi are mainly attributed to the polysaccharides with different molecular weights. Several mechanisms have been found to explain the anti-breast cancer effects of edible macro-fungi, such as inhibiting proliferation, inducing apoptosis and suppressing angiogenesis.

## 7. Cereals

Cereals are consumed worldwide, and rich in dietary fiber. A systematic review and meta-analysis of the evidence from prospective studies indicated an inverse association between cereal fiber intake and breast cancer risk (summary RR for the highest versus the lowest intake was 0.96, 95% CI = 0.90–1.02, *I*^2^ = 5%) [264].

Sorghum (*Sorghum bicolor*) is a primary cereal food in some parts of the world [265]. A study showed that sorghum suppressed tumor growth, induced cell cycle arrest, and inhibited metastasis via the Jak2/STAT pathway in nude mice bearing breast cancer xenografts [266]. Furthermore, 3-deoxyanthocyanin extracted from red sorghum bran exhibited cytotoxicity on MCF-7 cells with a CTC_50_ value of 300 µg/mL, and induced apoptosis mediated by upregulating the p53 gene and downregulating the Bcl-2 gene [267].

Barley (*Hordeum vulgare* L.) is widely consumed worldwide. A study showed that young barley (the grass of the barley plant) exhibited significant antiproliferative and proapoptotic activities in rat breast tumor model and in human breast cancer cells in vitro [268]. Wheat (*Triticum aestivum*) is a common kind of cereal, and contains rich nutritional constituents, such as starches and proteins (mainly in the endosperm), vitamins, minerals, phytochemicals and fibre (mainly in the wheat grain). A study showed that germinated wheat flour inhibited the growth of MCF-7 and MDA-MB-231 cells and induced apoptosis in vitro [269].

Collectively, sorghum, barley and wheat have shown the potential to inhibit the growth of breast cacer cells, mainly through inducing apoptosis and cell cycle arrest, and inhibiting metastasis.

## 8. Synergistic Effects of Dietary Natural Products with Anticancer Therapies

At present, chemotherapy and radiotherapy are frequently used in cancer treatment, but they are often accompanied with certain toxic adverse effects and drug resistance, which are common causes of chemotherapy failure and disease recurrence. Some dietary products and their bioactive components have shown synergistic effects with chemotherapy or radiotherapy, through enhancing their therapeutic effect or reducing side effects. For instance, combination of genistein and doxorubicin exerted a synergistic effect on MCF-7/Adr cells through stimulating the intracellular accumulation of doxorubicin and suppressing HER2/neu expression [270]. Besides, combination of genistein and centchroman (a selective estrogen receptor modulator) showed significantly higher cytotoxicity in human breast cancer cell lines compared to each drug used alone, and the nontumorigenic human mammary epithelial cell remained unaffected [271]. Moreover, equol enhanced the anticancer efficacy of tamoxifen in MCF-7 cells through inducing caspase-mediated apoptosis [272]. Equol could also be a potent radiosensitizer in both ER+ and ER− human breast cancer cells. It might be because equol enhanced cell death following irradiation and increased the DNA damage induced by remaining radiation, thereby reducing the surviving fraction of irradiated cells [273]. Furthermore, pomegranate extract enhanced tamoxifen-induced inhibition on cell viability of both sensitive and TAM-resistant MCF-7 cells by inducing cell death [274]. Meanwhile, DIM acted synergistically with Paclitaxel to inhibit growth of HER2/Neu human breast cancer cells via mediating the Her2/neu receptor and the downstream target ERK1/2. The cotreatment of DIM and Paclitaxel also enhanced apoptosis through the mitochondrial pathway (Bcl-2/PARP) [275]. Furthermore, DIM sensitized multidrug-resistant human breast cancer cells to γ-irradiation, judging from that G_2_/M phase cell cycle arrest was induced, intracellular ROS generation was increased and radiation-induced apoptosis was enhanced by DIM treatment (20 and 30 µM, 2 h before irradiation) [276]. Besides, the inactivation Akt/NF-κB signaling induced by DIM also contributed to sensitization of breast cancer cells to Taxotere-induced apoptosis [277]. TQ could also enhance the efficacy of other antitumor agents. Combination treatment of TQ and tamoxifen synergistically reduced cells viability and induced apoptosis in both ER+ MCF-7 and ER- MDA-MB-231 cell lines in vitro [278]. Besides, a TQ-Paclitaxel combination treatment inhibited breast cancer growth in cell culture and in mice, through the interplay with apoptosis network [279]. Furthermore, piperine could enhance the efficacy of TRAIL-based therapies for TNBC cells, possibly through the inhibition of survivin and activation of p65 phosphorylation [280]. Moreover, piperine sensitized TNBC cells to the cytotoxicity induced by gamma radiation [239]. In addition, a supercritical fluid extract of rosemary enhanced the therapeutic effect of 3 anti-breast cancer agents, tamoxifen, trastuzumab, and Paclitaxel [248]. Rice bran is one of the byproducts of rice milling. A team found that a modified arabinoxylan from rice bran increased the sensitivity of MCF-7 and HCC70 breast cancer cells to daunorubicin through enhancing the accumulation of daunorubicin in cancer cells [281]. Later, they found that the modified arabinoxylan was also an effective chemosensitizer to Paclitaxel, as evidenced by increased susceptibility of MCF-7 and 4T1 cells to Paclitaxel by over 100 folds. Mechanistically, the synergistical effects were through enhancing apoptosis and DNA damage, and inhibiting cell proliferation [282]. Furthermore, a study showed that wheat grass juice (squeezed from the mature sprouts of wheat seeds) taken by breast cancer patients during FAC chemotherapy (5-fluorouracil, doxorubicin, and cyclophosphamide combination) could reduce myelotoxicity and the dose, without decreasing efficacy of the chemotherapy [283].

**Figure 4:**
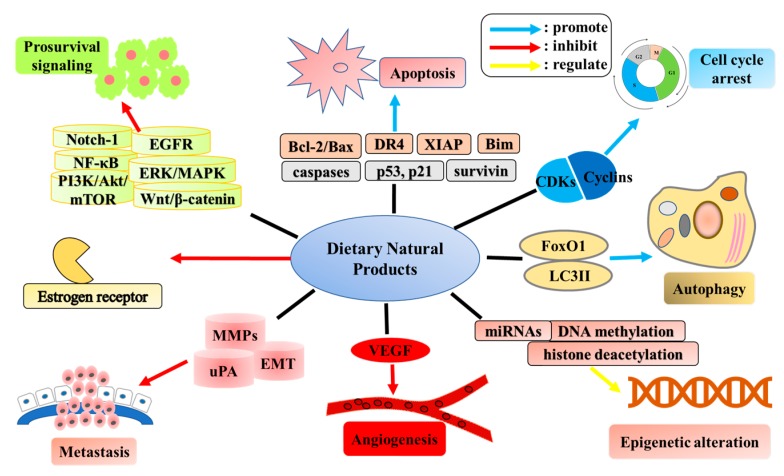
Mechanisms involved in the anti-breast cancer action of dietary natural products.

Finally, the epidemiological and experimental studies on dietary natural products for the prevention and treatment of breast cancer are summarized in Table 1 and Table 2, respectively. In addition, some effects of dietary natural products against breast cancer and possible mechanisms are shown in Figure 4.

## 9. Conclusions

The intake of some dietary natural products, such as soy, citrus fruits, cruciferous vegetables and mushrooms, is suggested to be inversely correlated with the risk of breast cancer by epidemiological studies. Furthermore, experimental studies also indicated that many dietary natural products could be potential sources for prevention and treatment of breast cancer. The following natural products and the corresponding bioactive components are noteworthy, including soy (genistein and daidzein), pomegranate (ellagitannins), mangosteen (mangostin), citrus fruits (naringin), apple (2α-hydroxyursolic), grape, mango, cruciferous vegetables (isothiocyanates), ginger (gingerols and shogaols), garlic (organosulfur compounds), black cumin (thymoquinone), edible macro-fungi (polysaccharides), and cereals. The anti-breast cancer effects of these natural products involve various mechanisms of action, such as inhibiting proliferation, migration, metastasis and angiogenesis of tumor cells, inducing apoptosis and cell cycle arrest, and sensitizing tumor cells to radiotherapy and chemotherapy. In the future, more anti-breast cancer bioactive compounds should be isolated and identified from dietary natural products, and more efforts should be made to assess the underlying mechanisms, potential toxicity and adverse effects. Moreover, the clinical efficacy of dietary natural products and their bioactive components on breast cancer patients needs to be further studied.

## Figures and Tables

**Table 1:** Epidemiological studies on association between natural product intake and breast cancer.

Natural Product	Study Type	Subject	Outcome	Association	Ref.
soy	cohort study	70,578 Chinese women aged 40–70 years	BC risk	overall: HR = 0.78, 95% CI = 0.63–0.97, premenopausal women: HR = 0.46; 95% CI: 0.29–0.74),	[45]
				ER+/PR+ postmenopausal women: HR = 0.72; 95% CI = 0.53–0.96	
				ER-/PR− premenopausal women: HR = 0.46; 95% CI = 0.22–0.97	
soy	cohort study	15,607 Japanese women aged 35 or above	BC risk	postmenopausal women:	[46]
				trend *p* = 0.023 for soy consumption;	
				trend *p* = 0.046 for isoflavone consumption	
soy	prospective study	649 Chinese women with BC	BC death	HR = 0.71, 95% CI = 0.52–0.98	[47]
			BC prognosis	ER+ patients: HR = 0.59, 95% CI = 0.40–0.93	
soy	cohort study	affected BC patients and unaffected high risk family members in Korea	BC risk	BRCA2 mutation carriers:	[48]
				HR = 0.39; 95% CI = 0.19– 0.79	
soy	cohort study	9514 BC survivors	risk of recurrence	HR = 0.75; 95% Cl = 0.61–0.92	[30]
soy	cohort study	3842 multiethnic women	all-cause mortality	no significant association	[50]
			BC specific mortality		
soy	cohort study	84,450 multiethnic women with BC	BC risk	no significant association	[51]
soy	cohort study	339 Korean women with BC	risk of recurrence	no significant association	[52]
Citrus fruits	meta-analysis	8393 participants: 3789 cases and 4,705 controls	BC risk	OR = 0.90; 95% CI = 0.85–0.96; *p* < 0.001	[112]
cruciferous vegetables	meta-analysis	18,673 BC cases	BC risk	RR = 0.85, 95% CI = 0.77–0.94	[150]
garlic	case-control study	285 Iranian women aged 25–65 years with BC	BC risk	ORs = 0.41, 95% CI = 0.20–0.83	[211]
mushroom	case-control study	362 women aged 30–65 years with BC	BC risk	postmenopausal women:	[255]
				for daily intake, OR=0.16, 95% CI = 0.04-0.54, *p*= 0.0058;	
				for average frequency, OR = 0.17, 95% CI = 0.05–0.54, *p* = 0.0037	
mushroom	meta-analysis	6890 BC cases	BC risk	premenopausal women:	[256]
				RR = 0.96, 95% CI = 0.91–1.00;	
				postmenopausal women:	
				RR = 0.94, 95% CI = 0.91–0.97	

BC, stands for breast cancer.

**Table 2:** The in vitro and in vivo effects of dietary natural products against breast cancer.

Natural Product	Constituents	Study Type	Main Effect and Possible Mechanism	Ref.
**Soy**				
soy	genistein	in vitro	- inducing cell cycle arrest, - improving mitochondrial functionality, - regulating oxidative stress, uncoupling proteins, antioxidant enzymes and sirtuin, - enhancing effects of anticancer drugs	[58,59,60]
soy	genistein	in vivo	reducing breast cancer incidence in a high-oestrogen environment	[61]
fermented doenjang	NA	in vitro	inducing cell cycle arrest, proliferation inhibition, and apoptosis	[64]
soy	genistein	in vitro	inducing apoptosis through: - downregulation of the cancerous inhibitor of protein phosphatase 2A - the inactivation of the IGF-1R/p-Akt signaling pathway	[65,66]
soy	6,7,4′-trihydroxyisoflavone	in vitro	inducing apoptosis and cell cycle arrest at *S*- and G_2_/M phases	[67]
soybean	NA	in vitro	inducing cell death via activation of caspase-3 and upregulation of proapoptotic molecule expression	[68]
soy	genistein	in vitro	inhibiting DNA methylation and increasing expression of tumor suppressor genes	[69]
soy	genistein	in vitro	inhibiting cancer cell growth through modulating the DNA damage response and cell cycle	[70]
soy	genistein	in vitro	inhibiting cancer cell growth through inhibiting activity of NF-κB via the Nocth-1 signaling pathway	[71]
soy	genistein	in vitro and in vivo	decreasing breast cancer stem-like cell population through Hedgehog pathway	[72]
soy	daidzein, equol	in vitro	inhibiting the invasion through the down-regulation of MMP-2 expression	[63]
**Fruits**				
pomegranate	NA	in vitro	inhibiting growth by inducing cell cycle arrest in G_2_/M and inducing apoptosis	[91]
pomegranate	NA	in vivo	preventing mammary tumorigenesis via concurrent disruption of ER and Wnt/-catenin signaling pathways	[92]
pomegranate	luteolin, ellagic acid, punicic acid	in vitro	inhibiting growth, increasing adhesion and decreasing migration of breast cancer cells	[94]
pomegranate	NA	in vitro and in vivo	showing cytotoxicities by targeting microRNAs155 and 27a, reducing cell proliferation and inducing apoptosis	[95,96]
pomegranate	ellagitannins, phenolic acids, conjugated octadecatrienoic acids	in vitro	inhibiting invasion and motility of cancer cells by inhibiting RhoC and RhoA protein expression	[97]
pomegranate	ellagitannin-derived compounds	in vitro	inhibiting aromatase activity and cell proliferation	[98]
pomegranate	NA	in vitro	inhibiting the cancerous lesion formation	[99,100]
mangosteen	NA	in vitro	inhibiting proliferation and inducing apoptosis	[101]
mangosteen	phenolics	in vitro	showing cytotoxicities	[102]
mangosteen	garcinone D, garcinone E, α-mangostin γ-mangostin	in vitro	dose-dependent anti-aromatase activity	[104]
mangosteen	α-mangostin	in vitro	inducing apoptosis through modulating HER2/PI3K/Akt and MAPK signaling pathways	[105]
mangosteen	α-mangostin	in vitro	inducing mitochondria-mediated apoptosis and cell cycle alterations	[106]
mangosteen	α-mangostin	in vitro	showing cytotoxicities	[107]
mangosteen	α-mangostin	in vitro	inhibiting FAS expression and activity, and inducing apoptosis	[108]
mangosteen	α-mangostin	in vitro	inducing apoptosis and decreasing the expression of ER alpha and pS2	[109]
mangosteen	α-mangostin	in vivo	increasing survival rates and suppressing tumor volume and the multiplicity of lymph node metastases	[110]
		in vitro	inducing apoptosis and cell cycle arrest	
mangosteen	panaxanthone	in vivo	suppressing tumor volumes and decreasing the multiplicity of lung metastasis and lymph node metastasis	[111]
		in vitro	inducing apoptosis	
Citrus fruit	polysaccharides	in vitro	inhibiting angiogenesis and cell migration	[113]
Citrus fruit	NA	in vitro	inducing apoptosis	[114]
Citrus fruit	NA	in vitro	inducing apoptosis via upregulating the expression of bax and caspase-3 genes and downregulating the expression of bcl-2 gene	[115]
Citrus fruit	naringin	in vitro	inhibiting growth potential by targeting β-catenin pathway	[116]
		in vivo	inhibiting cell proliferation and promoting cell apoptosis and G1 cycle arrest through modulating β-catenin pathway	
Citrus fruit	hesperidin	in vitro	anti-proliferative effect	[117]
apple	flavonoids	in vitro	inhibiting growth and inducing apoptosis	[118]
apple	polyphenol	in vitro	inhibiting tumorigenesis of pre-neoplastic cells by suppressing colony formation and ERK1/2 phosphorylation	[119]
apple	NA	in vitro	inhibiting proliferation and inducing cell cycle arrest at G_1_ phase	[120]
apple	2α-hydroxyursolic acid	in vitro	inhibit NF-κB activation through suppressing the proteasomal activities	[121,122]
apple	2α-hydroxyursolic acid	in vitro	antiproliferative and pro-apoptotic effect by regulating the p38/MAPK signal transduction pathway	[123]
apple	pectic acid	in vitro	inducing apoptosis and inhibiting cell growth	[124]
		in vivo	preventing tumor metastasis mice via over-expression of P53	
apple	NA	in vitro	enhancing the anti-proliferative effect of quercetin 3-beta-d-glucoside	[125]
grape	polyphenols	in vivo	inhibiting the lungs metastasis	[126]
		in vitro	inhibiting migration by blocking the PI3k/Akt and MAPK pathways	
grape	NA	in vitro	suppressing migration and invasion	[127]
grape	polyphenols	in vitro	inducing membrane damage, disrupting mitochondrial function and inducing G_2_/M cell cycle arrest	[128]
grape	amurensin G	in vitro	inhibiting VEGF production	[129]
grape	anthocyanin	in vitro	decreasing invasion, migration and bone turnover, via inhibiting expression of Snail and phosphorylated STAT3 and abrogating Snail-mediated CatL activity	[130]
mango	polyphenolics	in vitro	showing cytotoxic effects	[131]
		in vivo	reducing the tumor volume by regulating the PI3K/AKT pathway and miR-126	
mango	polyphenols	in vitro	inhibiting cell viability	[132]
mango	NA	in vitro	inducing apoptosis via the activation of oxidative stress	[133,134]
mango	pyrogallol	in vitro	inhibiting proliferation through mediating the AKT/mTOR signaling pathway	[135]
jujube	triterpenic acids	in vitro	inducing apoptotic cell death	[137]
jujube	betulinic acid	in vitro	inducing apoptosis through the mitochondria transduction pathway	[138]
jujube	NA	in vitro	inhibiting proliferation and inducing apoptosis	[139]
strawberry	NA	in vitro	showing cytotoxic effects	[140]
		in vivo	inhibiting the proliferation of tumor cells by activating apoptosis	
bilberry	NA	in vitro	inhibiting proliferation and inducing apoptosis	[141]
jamun fruit	NA	in vitro	inhibiting proliferation and inducing apoptosis	[142]
cranberry	NA	in vitro	inducing apoptosis and G1 phase arrest	[143]
peach	polyphenolics	in vivo	suppressing tumor growth and lung metastasis by inhibition of metalloproteinases gene expression	[144]
plum	phenolics and condensed tannins	in vitro	inducing apoptosis	[145]
quince fruit	NA	in vitro	inhibiting proliferation and invasiveness	[146]
graviola fruit	NA	in vitro	inhibiting the growth of cancer cells	[147]
		in vivo	inhibiting tumor growth by 32% (*p* < 0.01) through the EGFR/ERK signaling pathway	
litchi fruit	NA	in vitro	inhibited cell growth	[148]
		in vivo	reducing tumor mass volume	
pineapple	bromelain	in vitro	inducing apoptosis	[149]
**Vegetables**				
Cruciferous vegetables	benzyl isothiocyanate	in vitro	inducing apoptosis which was associated with: - inhibition of mitochondrial fusion - suppression of XIAP expression - generation of ROS	[155,156,157]
		in vitro and in vivo	suppressing the invasion and migration involving: - suppression of uPA activity and of Akt signaling - suppression on EMT process	[158,160]
		in vitro and in vivo	- inducing FoxO1-mediated autophagic death, - acting against the oncogenic effects of leptin, - suppressing proliferation and neovascularization, - inhibiting breast cancer stem cells	[161,162,163,164]
Cruciferous vegetables	phenethyl isothiocyanate	in vitro and in vivo	- inducing apoptosis, - suppressing adhesion, aggregation, migration and invasion, - prolonging the tumor-free survival and reducing the tumor incidence, - causing alterations in some genes	[165,166,167,168]
Cruciferous vegetables	sulforaphane	in vitro and in vivo	- downregulating ER-α expression, - inducing apoptosis, - inhibiting metastasis, - activating tumor suppressor Egr1, - downregulating telomerase, - regulating p38 MAPK and caspase-7 activations	[169,170,171,172,173,174,175]
Cruciferous vegetables	indole-3-carbinol	in vitro and in vivo	suppressing metastasis through - up-regulation of BRCA1 and E-cadherin/catenin complexes - suppressing EMT process and downregulating FAK expression - inhibition on MMP-2 expression - inhibiting CXCR4 and MMP-9 expression by downregulation of the NF-κB signaling pathway	[176,177,178,179]
		in vitro and in vivo	- regulating the cell cycle progression - downregulating expression of telomerase gene - inducing apoptotic cell death - suppressing the expression of IGF1R and IRS1 - inducing stress fibers and focal adhesion formation - stimulating expression of IFNγR1	[180,181,182,183,184,185,186,188]
Cruciferous vegetables	3,3′-diindolylmethane	in vitro	inducing apoptosis through: - inactivation of Akt and NF-κB activity - downregulating Bcl-2 and upregulating Bax	[189,190,191]
		in vitro	- inducing cell cycle arrest - lowering the invasive and metastatic potential - stimulating the expression and secretion of IFNγ	[192,193,194,195,196,197]
red beetroot	betanin	in vitro	showing a dose-dependent cytotoxic effect	[198]
**Spices**				
ginger	NA	in vitro	inhibiting the proliferation and colony formation	[200]
ginger	NA	in vitro	inducing apoptosis and inhibiting expression of c-Myc and hTERT	[201]
ginger	10-gingerol	in vitro	inhibiting proliferation and metastasis, inducing cell cycle arrest	[202]
ginger	6-gingerol	in vitro	inhibiting metastasis by suppressing MMP-2 and -9.	[203]
ginger	6-shogaol	in vitro	inhibiting invasion by reducing MMP-9 expression via blockade of NF-κB activation	[204]
			inhibiting invasion by suppressing invadopodium formation and MMP activity	[205]
			inhibiting growth and sustainability of spheroid generated from adherent breast cancer cells	[206]
ginger	6-dehydrogingerdione	in vitro	inducing apoptosis and cell cycle arrest in G_2_/M phase	[207]
garlic	diallyl disulfide	in vitro	inducing apoptosis though: - inhibition of histone deacetylation - inhibition of ERK and the activation of the SAPK/JNK and p38 pathways	[216,217]
		in vitro and in vivo	inhibit proliferation and metastasis via: - suppression of the SRC/Ras/ERK pathway - inactivation of the β-catenin signaling pathway	[218,219]
garlic	diallyl trisulfide	in vitro and in vivo	inducing apoptosis though: - overproduction of ROS and subsequent activation of JNK and AP-1 - upregulating FAS, Bax and p53, and down-regulating Akt and Bcl-2	[220,221]
		in vitro	- inhibiting migration and invasion; - inhibiting ER-α - decreasing protein level of FoxQ1	[213,222,223]
garlic	*S*-allyl mercaptocysteine	in vitro	inducing mitochondrial apoptosis and cell cycle arrest	[214]
garlic	allicin	in vitro	inhibiting invasion and metastasis	[215]
black cumin	extracts	in vitro	inducing apoptosis and inhibiting metastasis	[224,225]
black cumin	thymoquinone	in vitro and in vivo	inducing apoptosis through: - inhibiting Akt phosphorylation - inducing p38 phosphorylation via ROS generation	[228,229,230]
		in vitro	inhibiting proliferation by modulation of the PPAR-γ activation pathway	[231]
		in vitro	regulating COX-2 and E2	[232]
red chili pepper	capsaicin	in vitro and in vivo	- inducing apoptosis - inhibiting growth and migration	[233,234,235]
black pepper	piperine	in vitro and in vivo	- inhibiting growth, motility and metastasis - inducing apoptosis - suppressing the lung metastasis	[239,240,241]
saffron	crocetin	in vitro	inhibiting proliferation and invasion, through decreasing MMP expression	[243]
clove	eugenol	in vitro and in vivo	inhibiting growth and proliferation, inducing apoptosis through targeting the E2F1/survivin pathway	[246,247]
rosemary	extracts	in vitro	exerting antitumor activity through mediation of ER-α and HER2 signalings	[248]
wasabi	6-(methylsulfinyl)hexyl isothiocyanate	in vivo	inducing apoptosis by inhibiting NF-κB and regulating the PI3K/AKT pathway	[249]
coriander	root extract	in vitro	affecting antioxidant enzymes, inducing G_2_/M phase arrest and apoptosis	[250]
**Edible Macro-Fungi**				
Antrodia camphorate	methyl antcinate A	in vitro	suppressing the population of cancer stem-like cells	[257]
*Trametes robiniophila* Murr.	polysaccharides	in vitro	induced apoptosis through down-regulation of metadherin	[258]
*Pleurotus abalonus*	polysaccharides	in vitro	inhibiting antiproliferation and inducing apoptosis via ROS-mediated mitochondrial apoptotic pathway	[259]
*Ganoderma lucidum*	extracts	in vitro	inhibiting invasion via inhibiting the expression of uPA and uPA receptor	[260]
			causing both apoptosis and necrosis	[261]
*Agaricus bisporus*	extracts	in vitro	suppressing the aromatase activity dose-dependently	[262]
*Pleutorus eous*	polysaccharides	in vitro	inhibiting angiogenesis and inducing apoptosis	[263]
**Cereals**				
Sorghum	extracts	in vivo	suppressing tumor growth, inducing cell cycle arrest, and inhibiting metastasis	[266]
	3-deoxyanthocyanin	in vitro	inducing apoptosis by upregulating the p53 gene and downregulating the Bcl-2 gene	[267]
barley	extracts	in vitro and in vivo	exerting antiproliferative and pro-apoptotic activities	[268]
wheat	germinated wheat flour	in vitro	inhibiting growth and inducing apoptosis	[269]

## Abbreviations

The following abbreviations are used in this manuscript: AP-1: active protein-1; ATF-2: activating transcription factor 2; Bax: Bcl-2-associated X protein; Bcl-2: B-cell lymphoma 2; Bcl-xL: B-cell lymphoma-xL; Bim: B-cell lymphoma 2 interacting mediator of cell death; BITC: benzyl isothiocyanate; CDKs: cyclin-dependent kinases; CXCR4: C-X-C chemokine receptor type 4; DADS: diallyl disulfide; DATS: diallyl trisulfide; DR4: death receptor 4; EGFR: epidermal growth factor receptor; EMT: epithelial-mesenchymal transition ER: estrogen receptor; ERK: extracellular regulated protein kinase; FAK: focal adhesion kinase; FAS: fatty acid synthase; FFQ: food frequency questionnaire; FoxQ1: Forkhead Box Q1; GSK-3β: glycogen synthase kinase 3β; HER2: human epidermal growth factor receptor 2; HIF: hypoxia-inducible factor; hTERT: human telomerase reserve transcriptase; I3C: indole-3-carbinol; IFNγ: interferon gamma; IGF1R: insulin-like growth factor-1 receptor; IL-2: interleukin 2; IRS1: insulin receptor substrate-1; Jak2: Janus kinase; JNK: c-Jun *N*-terminal kinase; MAA: methyl antcinate A; MAPK: mitogen-activated protein kinase; MMP: matrix metalloproteinase; mTOR: mechanistic target of rapamycin; NF-κB: nuclear factor kappa-light-chain-enhancer of activated B cells; NMU: *N*-methyl nitrosourea; PARP: poly-ADP-ribose polymerase; PCNA: proliferating cell nuclear antigen; PEITC: phenethyl isothiocyanate; PI3K: phosphatidylinositol 3-kinase; Pin1: Peptidyl-prolyl cis-trans isomerase; PR: progesterone receptor; PUMA: p53 upregulated modulator of apoptosis; RhoA: Ras homolog gene family, member A; RhoC: Ras homolog gene family, member C; ROS: reactive oxygen species; SAMC: S-allyl mercaptocysteine; SAPK: stress-activated protein kinase; SFN: sulforaphane; STAT3: signal transducers and activators of transcription 3; TNBC: triple-negative breast cancer; TNF-α: tumor necrosis factor α; TQ: thymoquinone; TRAIL: TNF related apoptosis inducing ligand; uPA: urokinase-type plasminogen activator; VCAM-1: vascular cell adhesion molecule 1; VEGF: vascular endothelial growth factor; XIAP: X-linked inhibitor of apoptosis.
